# Nutritional and genetic variation in a core set of Ethiopian Tef (*Eragrostis tef*) varieties

**DOI:** 10.1186/s12870-022-03595-9

**Published:** 2022-04-28

**Authors:** Nelzo C. Ereful, Huw Jones, Nick Fradgley, Lesley Boyd, Hirut Assaye Cherie, Matthew J. Milner

**Affiliations:** 1 NIAB, 93 Lawrence Weaver Road, Cambridge, CB3 0LE UK; 2 Philippine Genome Centre, University of the Philippines Los Baňos, Laguna, Philippines; 3 Department of Plant Sciences, University of Cambridge, Downing Street, Cambridge, CB2 3EA UK; 4 Faculty of Chemical and Food Engineering, Bahir Dar Institute of Technology, P.O.Box 26, Bahir Dar, Ethiopia

## Abstract

**Background:**

Tef (*Eragrostis tef*) is a tropical cereal domesticated and grown in the Ethiopian highlands, where it has been a staple food of Ethiopians for many centuries. Food insecurity and nutrient deficiencies are major problems in the country, so breeding for enhanced nutritional traits, such as Zn content, could help to alleviate problems with malnutrition.

**Results:**

To understand the breeding potential of nutritional traits in tef a core set of 24 varieties were sequenced and their mineral content, levels of phytate and protein, as well as a number of nutritionally valuable phenolic compounds measured in grain. Significant variation in all these traits was found between varieties. Genome wide sequencing of the 24 tef varieties revealed 3,193,582 unique SNPs and 897,272 unique INDELs relative to the tef reference var. Dabbi. Sequence analysis of two key transporter families involved in the uptake and transport of Zn by the plant led to the identification of 32 Zinc Iron Permease (ZIP) transporters and 14 Heavy Metal Associated (HMA) transporters in tef. Further analysis identified numerous variants, of which 14.6% of *EtZIP* and 12.4% of *EtHMA* variants were non-synonymous changes. Analysis of a key enzyme in flavanol synthesis, flavonoid 3′-hydroxylase (F3’H), identified a T-G variant in the tef homologue Et_s3159-0.29-1.mrna1 that was associated with the differences observed in kaempferol glycoside and quercetin glycoside levels.

**Conclusion:**

Wide genetic and phenotypic variation was found in 24 Ethiopian tef varieties which would allow for breeding gains in many nutritional traits of importance to human health.

**Supplementary Information:**

The online version contains supplementary material available at 10.1186/s12870-022-03595-9.

## Background

Tef (*Eragrostis tef*) is a tropical cereal that has its origins in the Ethiopian highlands, where it was domesticated and has been grown for thousands of years [1]. Globally tef is a minor cereal crop in terms of both production and planted area, with Ethiopia growing an estimated 90% of the annual global tef crop on about three million hectares, which equates to about a quarter of the Ethiopian grain-cultivated area [2]. Compared to other cereals tef is considered a resilient crop. It can withstand adverse weather conditions, growing well at elevations between 1800 and 2200 m above sea level, in regions where there is adequate rainfall [3]. Tef is a staple of Ethiopian diets, providing 11% of the per capita caloric intake and two-thirds of the average Ethiopians daily protein [4].

Tef is widely considered a healthy alternative to cereals such as wheat, maize and rice as it does not contain gluten, is high in slowly digestible starch, rich in calcium (Ca) and polyphenols [5, 6]. However, levels of zinc (Zn) in the grain (in the range of 28–40 mg/kg) are often less than the recommended level of 40 mg/kg necessary to meet human nutritional requirements [1, 7, 8], with further fortification efforts being hampered by low Zn levels in many Ethiopian soils [1].

While breeding efforts to improve tef have been ongoing in Ethiopia since the mid-1950s germplasm advancements have been slow, with only 42 new tef varieties being released [3, 9, 10]. A better understanding of tef nutritional diversity and its genetic control could help drive nutritional gains for this crop. Recent advancements in our understanding of the genetics of tef include a fairly complete genome assembly, the release of gene models, as well as a number of RNASeq datasets which are all publicly available [11]*.* These allow for translation of knowledge on grain fortification from other species, supporting more rapid advancements in efforts to improve the nutritional potential of tef [12–16].

Several gene families have been identified that support accumulation and transport of various heavy metals within the plant. The Zinc Iron Permease (ZIP) family of transporters, the Heavy Metal Associated (HMA) and Metal Tolerance Protein (MTP) families, and the Natural Resistance-Associated Macrophage protein (NRAMPs) family of transporters have all been shown to be integral in micronutrient transport in plants [17–21]. The transport of Zn from soil to seed can involve a number of these transporters, with Zn primarily being accumulated within the seed embryo and aleurone layer [22]. In rice Zn uptake from the soil is thought to occur through the transporters *OsZIP5* and *OsZIP9* [23, 24]. Others have suggested that many routes may be available to Zn uptake, as no single transporter is believed to be solely responsible for the uptake of Zn from the soil [25–29]. Several transporter families have also been implicated in the transport of Zn through the plant to the developing grains [18, 30, 31]. These include the HMA family and the ZIP family of transporters, both shown to be able to transport a broad range of metals [18, 19, 26, 32], which can also include the transport of unwanted metals such as cadmium (Cd) and lead (Pb), both of which are detrimental to most plants and animals [17, 19, 26, 32–34].

Other factors can also influence the Zn content in both plant leaves and grain. These include phytates, which readily bind cations such as calcium (Ca), iron (Fe) and Zn, phosphate (P) and phytate being found to correlate with Zn concentration in the grain of both wheat and rice [31, 35]. In wheat, nitrogen (N) content has been found to positively correlate with Zn content in the grain [8]. Recent work has shown that macronutrients such as N, P and sulphur (S), can also influence overall micronutrient content [36]. Thus, a holistic approach to nutrient enhancement is required to ensure adequate Zn supply in the human diet.

In this study we set out to understand the phenotypic variation of several nutritional traits in tef varieties commonly grown and consumed in Ethiopia. We used whole genome sequencing and single nucleotide polymorphisms (SNPs) to determine the genetic relationship between these tef varieties. This genomic data was then used to look for relationships between the tef varieties and the nutritional traits to identify those varieties with optimal nutritional potential for breeding. In addition, SNPs within candidate genes underlying nutritional traits of interest were examined to determine linkages with the trait variation.

## Materials and methods

### Plant material and flour preparation

Twenty-four tef varieties (Additional file 1) were sown during the main cropping season in July 2018, at a seeding rate of 15 kg per ha, at the experimental station of Adet Agricultural Research Center, Ethiopia. Adet (11 o28′ N, 37 o48′ E; 2216 m a.s.l) is located 42 km southwest of Bahir Dar, the capital city of the Amhara regional state, Ethiopia. Agronomic and cultural practices used were those recommended for tef production, and included application of 40 kg N and 60 kg P_2_O_5_ per ha [37]. The soil at Adet is brown Nitosol. The 24 tef varieties used in this study are publicly available in Ethiopia from the Adet Agricultural Research Center. The voucher specimen ID numbers are listed in Additional file 1.

Whole tef grains (20 g) of each variety were milled to flour using a Laboratory Hammer Mill (Model: TPS-JXFM110, China) to a sieve size of 0.5 mm, packed in polyethylene bags and stored at room temperature until analysed.

### Analyses of nutritional traits

Analyses of all nutritional traits were undertaken at NIAB, UK. Analyses included assessment of elemental concentrations, phenolic compounds, phytate and N content.

Elemental concentrations were assessed using inductive coupled plasma mass spectrometry (ICP-MS). ICP-MS was used to detect the beneficial micronutrients Ca, Cu, Fe, K, Mg, Mn, Mo, P, S, Se, Ti, and Zn, and the detrimental elements Cd and Pb. Approximately 0.2–0.3 g of flour was digested in 5 mL of nitric acid (Sigma) overnight in a 7 mL bijou. The digested sample was transferred to a 50 mL beaker and heated to 115 °C to remove the residual acid, after which 3 mL of H_2_O_2_ (Sigma) was added. After H_2_O_2_ reduction the remaining powder was dissolved in 15 mL of ddH_2_0 and samples analysed on the ICP-MS using a Thermo-Fisher Scientific iCAP-Q equipped with CCTED (collision cell technology with energy discrimination). Three independent technical replications were run for each tef flour sample. Possible soil contamination was identified where flour samples had greater than 100 mg/kg of Fe, Al and possibly Si, and/ or Ti above 1 μg/g [38, 39].

N content was determined using Dumas analysis. Flour samples were dried at 104 °C for 3 h. One gram of flour was loaded according to the manufacturer’s instructions (Leco TruMacN Dumas gas analyser) to determine N content. Dumas gas analysis was performed on three, technical replicate 1 g aliquots of flour from each variety.

Phenolic compounds were assessed using HPLC. Flour samples of approx. 2.5 g were extracted into 50 ml of ethanol-acetic acid (10% 1 M acetic acid v/v) under reflux conditions for 2 h. Extracts were stored at − 20 °C until analysed. The extracts were prepared for chromatography by centrifugation for 2 min at 13000 rpm, then filtrated through a 0.2 μm filter. The compounds were separated using the Dionex Ultimate 3000 HPLC system. A 150 mm × 4.6 mm × 5 μm × 100 Å Kinetix C18 column was used, with a gradient 0.1% formic acid /acetonitrile mobile phase running at 0.2 ml/minute (gradient of 0.95: 0.05 for 2 min, then 0.72; 0.28 for 18 min, 0.00; 0.10 for 28 min, and then held until 45 min) were used. The column effluent was monitored with a PDA detector between 200 and 600 nm, with data recorded at 254 nm, 280 nm, 340 nm and 520 nm.

Phytate was measured using the commercial Megazyme Phytic Acid Assay Kit (Brey, Ireland) following the manufacturer’s instructions with minor modifications. Approximately 100 mg of flour was digested in 1.8 mL HCl (0.66 M) in 2.2 mL tubes, placed in a rotator mixer overnight with a constant rpm of 20, at room temperature. Three technical replicates were applied for each flour sample.

### Genomic DNA isolation and sequencing

DNA was extracted from flour of the 24 tef varieties using Qiagen’s DNAeasy Kit as per the manufacturer’s instructions, including RNase treatment. The same flour samples were used for DNA extraction as were used of nutrient trait analyses. DNA was shipped to Novogene (Cambridge UK) for Illumina Sequencing. Illumina libraries contained on average 350 bp inserts and were sequenced using paired end technology. The estimated genome coverage of each tef variety was at least 25X, based on an estimated size of the genome of 622 MB [11]. Raw reads for all tef varieties tested have been deposited with the ENA under the ArrayExpress accession E-MTAB-8827.

### Whole genome sequence assembly and variant calling

Paired-end sequence reads were provided by the sequencing service that had already undergone quality checks, including the removal of over-represented sequences, adapters, and reads with low-quality base scores (at Q > 20). No further analysis using FastQC [40] was therefore required. The paired-end reads were mapped against the indexed reference sequence of the tef variety Dabbi using BWA mem [11, 41]. Output was piped to bam and sorted using SAMtools. PICARD tool was used to assign reads to a single Read Group (option: AddOrReplaceReadGroup). PCR duplicates were flagged using PICARD (option: MarkDuplicates). A dictionary for the reference genome of contig names and sizes was also created using PICARD (option: CreateSequenceDictionary). Variants were identified using the Broad Institute Genome Analysis Tool Kit (GATK4 [42];). GATK (option: Haplotypecaller) was run for each tef variety, using its default parameters, to call variants between the reference Dabbi genome sequence and the variety sequence in both Variant Call Format (VCF) and Genomic VCF (option: –ERC GVCF) modes. Only variants with a quality score of > 20 were considered. The variant SNPs were then analysed using gff3 and bcftools csq [43] to determine whether the SNP was within a coding region, and whether the SNP was synonymous vs non-synonymous.

Using PLINK2 the VCF file was converted into PLINK binary format (bed, bim and fam). A phylogenetic tree was created using SNPhylo and the biallelic SNPs identified across the 24 tef varieties [44]. A principal co-ordinate analysis (PCA) plot was constructed using the same biallelic SNPs and analysed using the R program SNPrelate [45]. FastStructure (with logistic prior, K = 5) was used to infer population structure within the 24 tef varieties using the same SNPs [46].

### Identification of zinc iron permease and heavy metal associated transporter family members in tef

The *Eragrostis tef* genome was searched using the Biomart tool in Ensembl plants (http://plants.ensembl.org/biomart/martview/) to identify predicted genes that contain both the PFAM domain PF02535 and Interpro ID IPR003689, or Interpro ID IPR027256. The PFAM domain PF02535 and Interpro ID IPR003689 are used for the identification of ZIP transporter proteins and Interpro ID IPR027256 for identification of HMA transporter proteins. Candidate ZIP transporter and HMA transporter proteins in *Arabidopsis* and rice were also identified using Ensembl’s Biomart. The amino acid sequences of these candidate genes were aligned using MUSCLE as part of MEGA X [47] using the default parameters and a phytogenic tree created using the Maximum Likelihood option, with 50 replications used for the determination of bootstrap values. Reciprocal BLAST, using the amino acid sequence of the rice ZIP and HMA genes as the query, was further used to identify putative homologues of each gene in tef using CoGe (https://genomevolution.org/coge/CoGeBlast.pl).

### Statistical analysis

Micronutrient, N and phytate levels were tested for normality and homogeneity of variance using Shapiro-Wilk’s test in R, which indicated a normal distribution. Two-way analysis of variance (ANOVA) was used to infer significance between tef varieties using the aov function and TukeyHSD commands in R. A least significant difference of 5% probability level was used as a post-hoc test to determine significance. Results were plotted using R ggplot2 and ggpubr packages [48, 49]. Correlations between micronutrients, N and phytate were calculated in R using the Spearman rank correlation (cor.test) or Kruskal-Wallis test for categorical variables [45].

All statistical analyses of phenolic compound levels were performed using Genstat v.16 (VSN International 2020). The levels of the phenolic compounds were analysed using a modified two-way ANOVA approach, General Linear Regression. The model applied was replicate by variety. Only comparisons having a F probability < 0.001 were considered as statistically significant. The linear relationship between phenolic compound levels were measured using the Pearson correlation coefficient in Excel (Microsoft). Boxplots were generated using R ggplot2.

## Results

### Comparison of nutritional traits in flour of twenty-four tef varieties

**Fig. 1:**
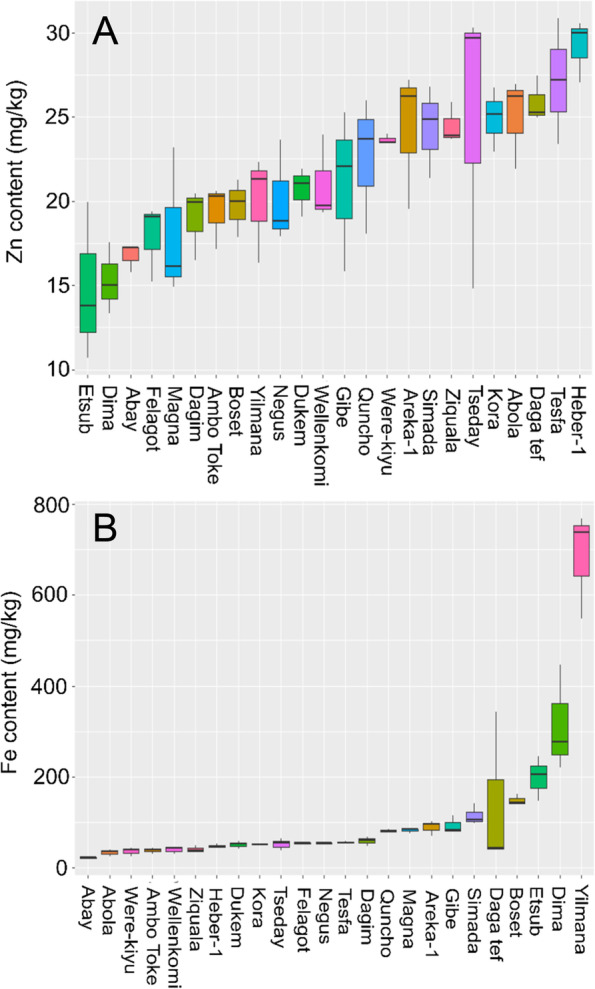
Zn and Fe levels in flour of 24 tef varieties grown at the Adet Research Agricultural Research Center in 2018/19. The box defines the upper and lower quartiles. The lines extending vertically, “whiskers”, indicate variability outside the upper and lower quartiles

**Fig. 2:**
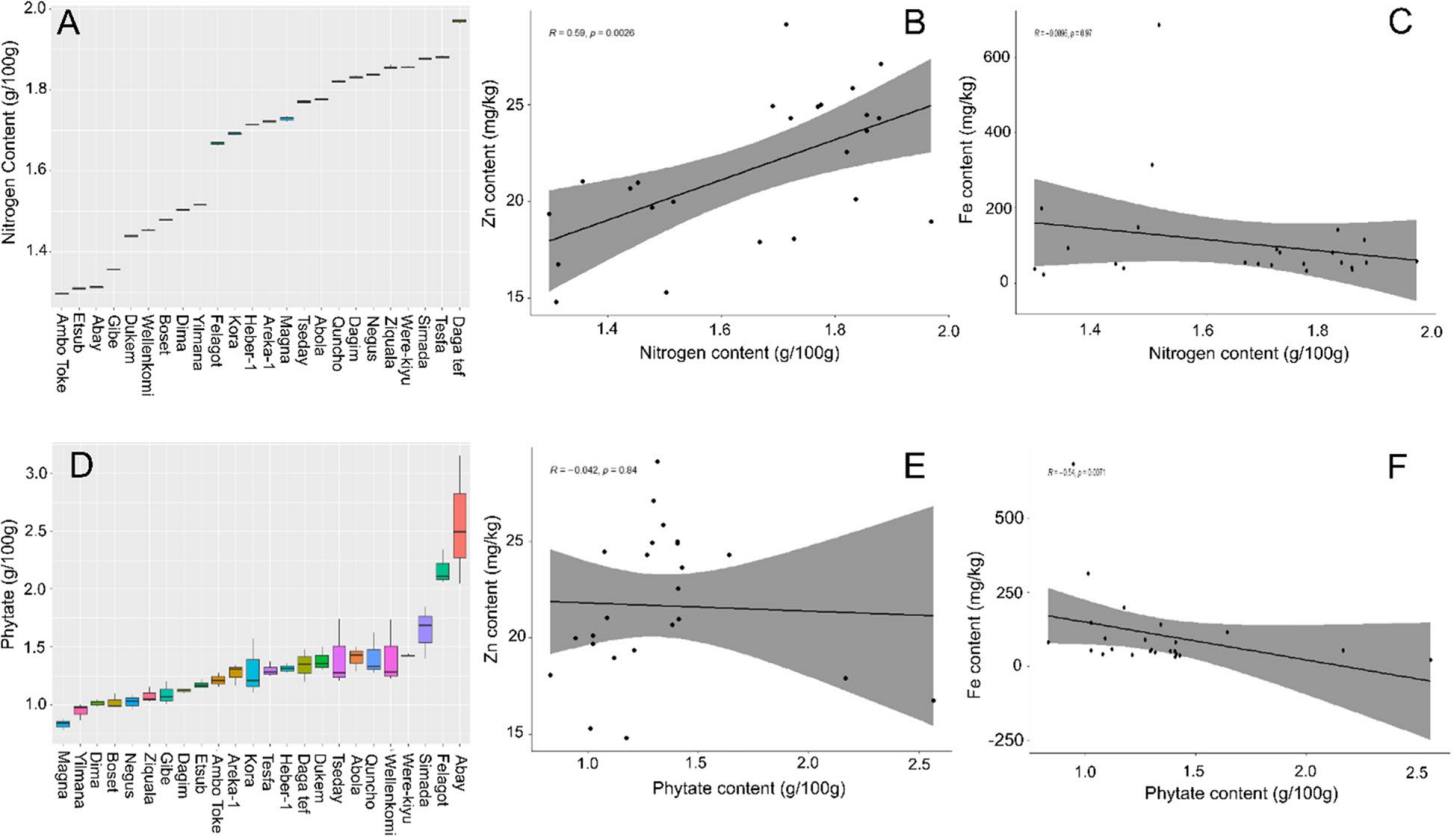
Relationship between Zn, Fe, nitrogen and phytate levels in 24 tef varieties. Nitrogen levels (**A**). Correlation between Zn and nitrogen content (**B**). Correlation between Fe and nitrogen content (**C**). Phytate levels (**D**). Correlation between Zn and phytate content (**E**). Correlation between Fe and phytate content (**F**)

To understand the nutritional potential of 24 tef varieties (Additional file 1) currently grown and consumed in Ethiopia we measured the levels of a number of nutritional traits, comparing the relationship between these traits, and using genomic data to look at the genetic relationship between these 24 tef varieties relative to these nutritional traits. The nutritional traits included elemental micronutrient (Fig. 1 & Additional file 2), nitrogen as a proxy for protein content (Fig. 2A), phytate (Fig. 2D) and a range of phenolic compounds (Additional file 3).

Significant variation between the 24 tef varieties was found for all micronutrients tested (Additional file 2) (p.val < 0.001). Focus on elements essential for human health, Zn and Fe (Fig. 1), showed Zn flour concentrations to range from 14.8 (var Etsub) to 29.2 mg/kg (var. Heber-1) and Fe to range from 22.6 (var Abay) to 684.25 mg/kg (var Yilmana) (Fig. 1A & B). Positive correlations were found between Zn and Ca, Mg, P, S, and to a lesser extent K, while negative correlations were found with Cu, Fe, Mo and Se. A positive correlation was also seen between Zn and Cd (Suppl. Fig. 1). No correlations were found between Pb or Ti and the other elements measured.

Cd is a toxic micronutrient, detrimental to human health. High levels of Cd were seen in many of the tef samples tested, the highest levels of Cd being found in the variety Wellenkomi, having 40.7 μg/kg. Only one of the tef varieties, var. Magna, had Cd levels below the current EU limits for Cd in food products, being below 1 μg/kg (1 ppm) [50].

The N levels within the tef flours were measured as a proxy for protein content [51]. Significant differences were found between the tef varieties, with N values ranging from 1.3 g/100 g in var. Ambo Toke to nearly 2 g/100 g in var. Dagan tef (p val < 0.001) (Fig. 2A). Using the standard conversion of 5.95 this would give protein content in the range of 7.79 to 11.71% [52]. A positive correlation was found between Zn content and N levels in the flours tested (*R* = 0.59; p val. 0.01; Fig. 2B) but not for Fe and N (*R* = 0.0096; *p* val 0.97; Fig. 2C).

Significant differences were found in phytate levels between the 24 tef varieties (*p* val < 0.01) (Fig. 2D). The phytate levels ranged from 0.83 g/100 g in var. Magna to 2.56 g/100 g in var. Abay. No significant correlations were observed between phytate and Zn (R 0.042 *p* val. 0.84; Fig. 2E), but a significant negative correlation was found between Fe and phytate (*R* − 0.54 p val. 0.007; Fig. 2F). There was no significant correlation between overall P levels, measured via ICP-MS, and phytate (*R* = 0.29; p val. 0.18).

Flour of the tef varieties were also screened for 19 phenolic compounds (Additional file 3). Significant differences (F *p* val. < 0.001) were found between the 24 tef varieties for 11 phenolic compounds, significant differences between tef varieties not being found for gallic acid. Seven phenolic compounds were not detected. These include cyanidin, cyanidin glycoside, delphinidin, delphinidin glycoside, flavone, pelagonidin and p-coumaric acid.

**Fig. 3:**
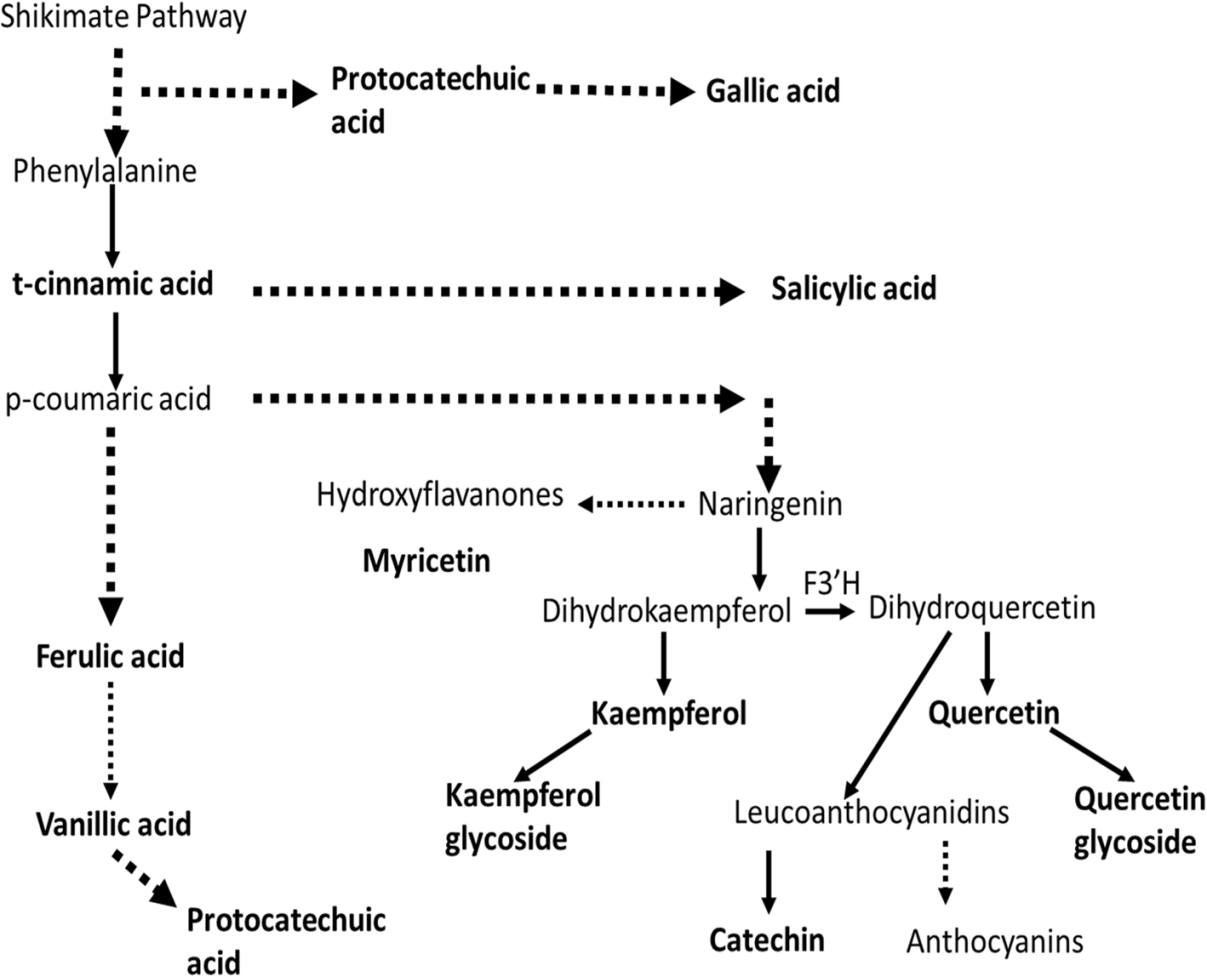
Biochemical pathway of selected phenolic compounds. The schematic shows the biochemical pathway relationship between the phenolic compounds measured in this study. Flavonoid 3′-hydroxylase (F3’H) is a key enzyme in the conversion of dihydrokaempferol to dihydroquercetin

**Fig. 4:**
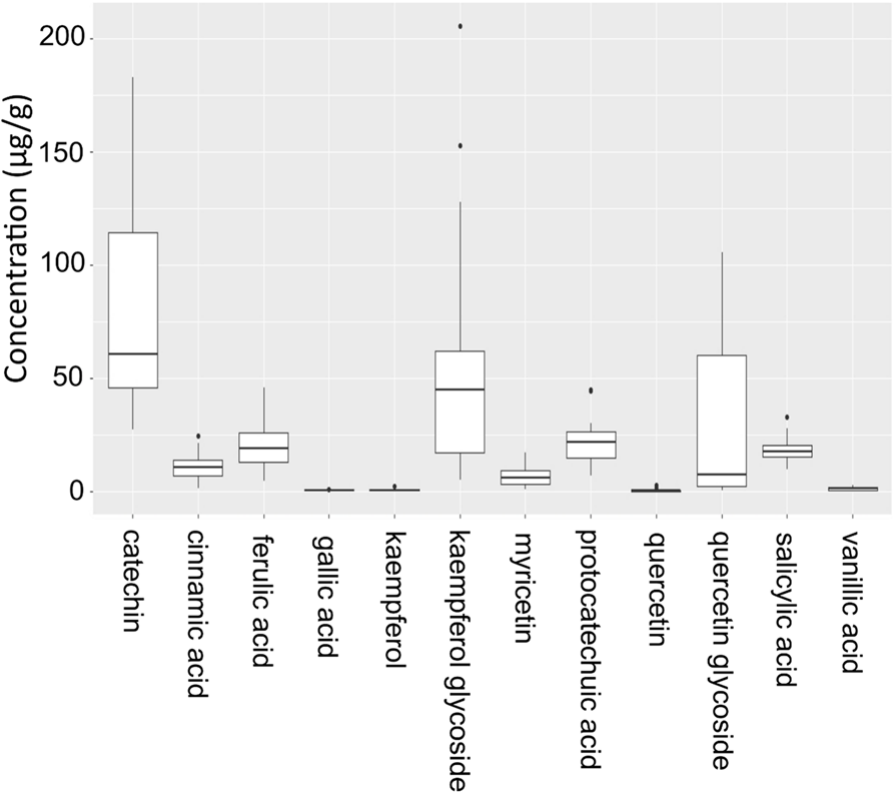
Levels of 12 phenols identified in the flours of 24 tef varieties. Each box shows the range of concentrations in μg/g of 12 phenolic compounds measured in the 24 tef varieties. The box defines the upper and lower quartiles. The lines extending vertically, “whiskers”, indicate variability outside the upper and lower quartiles, with points outside those lines, represented by dots, being considered outliers

Kaempferol, quercetin, catechin and myricetin all belong to a group of phenolic compounds commonly known as flavonoids (Fig. 3) [53]. The levels of kaempferol and quercetin were low in all the tef varieties, the majority of these compounds being present as kaempferol glycoside and quercetin glycoside (Fig. 4). The levels of kaempferol glycoside and quercetin glycoside ranged from 4.92 (var. Tseday) to 205.79 μg/g (var. Magna), and 0.46 (var.Felagot) to 105.41 μg/g (var. Quncho), respectively. The levels of catechin ranged from 27.41 (var. Yilmana) to 183.19 μg/g (var. Tseday) (Fig. 4). Catechin is derived from dihydroxyquercetin (Fig. 3) and a precursor of proanthocyanidins, which are thought to give rice its red colouration [53]. The levels of myricetin ranged from 0.82 (var. Were-kiyu) to 17.24 μg/g (var. Dima) (Fig. 4).

The levels of *t*-cinnamic acid ranged from 1.51 μg/g in var. Baset to 24.65 μg/g in var. Dukem. Cinnamic acid is a precursor of ferulic acid, which in turn gives rise to vanillic acid. A strong positive correlation was found between cinnamic and ferulic acids, and a lesser positive correlation with vanillic acid. However, the levels of vanillic acid were low in all 24 tef varieties (Additional file 3, Additional file 4).

Protocatechuic acid and gallic acid are synthesised from a side branch of the shikimate pathway that leads to the synthesis of folates and aromatic amino acids, including phenylalanine (Fig. 3 [53];). The levels of protocatechuic acid ranged from 6.78 μg/g (var. Areka-1) to 44.78 μg/g (var. Felagot) (Additional file 3). The levels of gallic acid were low in all 24 tef varieties, ranging from 0.12 μg/g (var. Dima) to 0.97 μg/g (var. Ambo take (Fig. 4).

Pearson’s correlation analyses were undertaken on the levels of each phenolic compound, phytate and Fe (Additional file 4). A strong, positive correlation was seen between cinnamic acid and ferulic acid (*r* = 0.78), fitting with the biochemical pathway (Fig. 3), where cinnamic acid is a precursor of ferulic acid. Similarly, a positive association was seen between ferulic acid and vanillic acid (*r* = 0.51), vanillic acid sitting downstream of ferulic acid. However, positive correlations were also seen between ferulic acid and myricetin (*r* = 0.61), which may relate to their common precursor cinnamic acid, and between cinnamic acid and quercetin (*r* = 0.49) and quercetin glycoside (*r* = 0.50).

The precursors of kaempferol and quercetin, dihydrokaempferol and dihydroquercetin, exist in an equilibrium controlled by a flavonoid 3′-hydroxylase (F3’H) enzyme which converts dihydrokaempferol to dihydroquercetin. This resulted in negative correlations between kaempferol and quercetin (*r* = − 0.28) and kaempferol glycoside and quercetin glycoside (*r* = − 0.43); the majority of kaempferol and quercetin being present in the glycosylated state. This was reflected in positive correlations between kaempferol and quercetin glycoside (*r* = 0.62) and quercetin and kaempferol glycoside (*r* = 0.45).

Catechin is derived from dihydroquercetin and therefore competes for synthesis with quercetin. Consequently, a negative correlation was observed between the levels of catechin and quercetin (*r* = − 0.32). However, a positive correlation was seen between catechin and quercetin glycoside (*r* = 0.52). A positive correlation was also observed between catechin and kaempferol (*r* = 0.57) and a negative correlation with kaempferol glycoside (*r* = − 0.51).

In addition to positive correlations with kaempferol (*r* = 0.62) and catechin (*r* = 0.52), positive correlations were observed between quercetin glycoside and other phenolic compounds, including ferulic acid (*r* = 0.65), myricetin (*r* = 0.56), cinnamic acid (*r* = 0.50). These correlations may indicate a positive feedback mechanism operating through the biosynthetic pathways leading to quercetin glycoside synthesis.

A negative correlation was found between gallic acid and salicylic acid (*r* = − 0.43), and between catechin and protocatechuic acid (*r* = − 0.44), while a positive correlation was observed between protocatechuic acid and Fe content (*r* = 0.40). No significant correlations were found between phytate levels and any of the phenolic compounds measured in this study (Additional file 4).

### Genetic relationship between the twenty-four tef varieties

All 24 tef varieties were sequenced to greater than 25X coverage. Sequences were compared to the reference var. Dabbi across the whole genome. The varieties differed by as few as 1.567 million SNPs in var. Areka-1 relative to Dabbi, to as high as 2.372 million SNPs in var. Yilmana (Additional file 5). There was also considerable variation in INDELs, with Yilmana showing the fewest INDELs at 343,257 and Wellenkomi the highest at 520,234. Overall, 3,193,582 unique SNPs and 897,272 unique INDELs were found, containing a minimum allele frequency of 0.1 within the 24 varieties tested. Considering that the whole tef genome is estimated to be 622 Mb in size, the 2.372 million variants identified in var. Yilmana relative to the reference var. Dabbi, equates to roughly four variants in every kb of the genome.

**Fig. 5:**
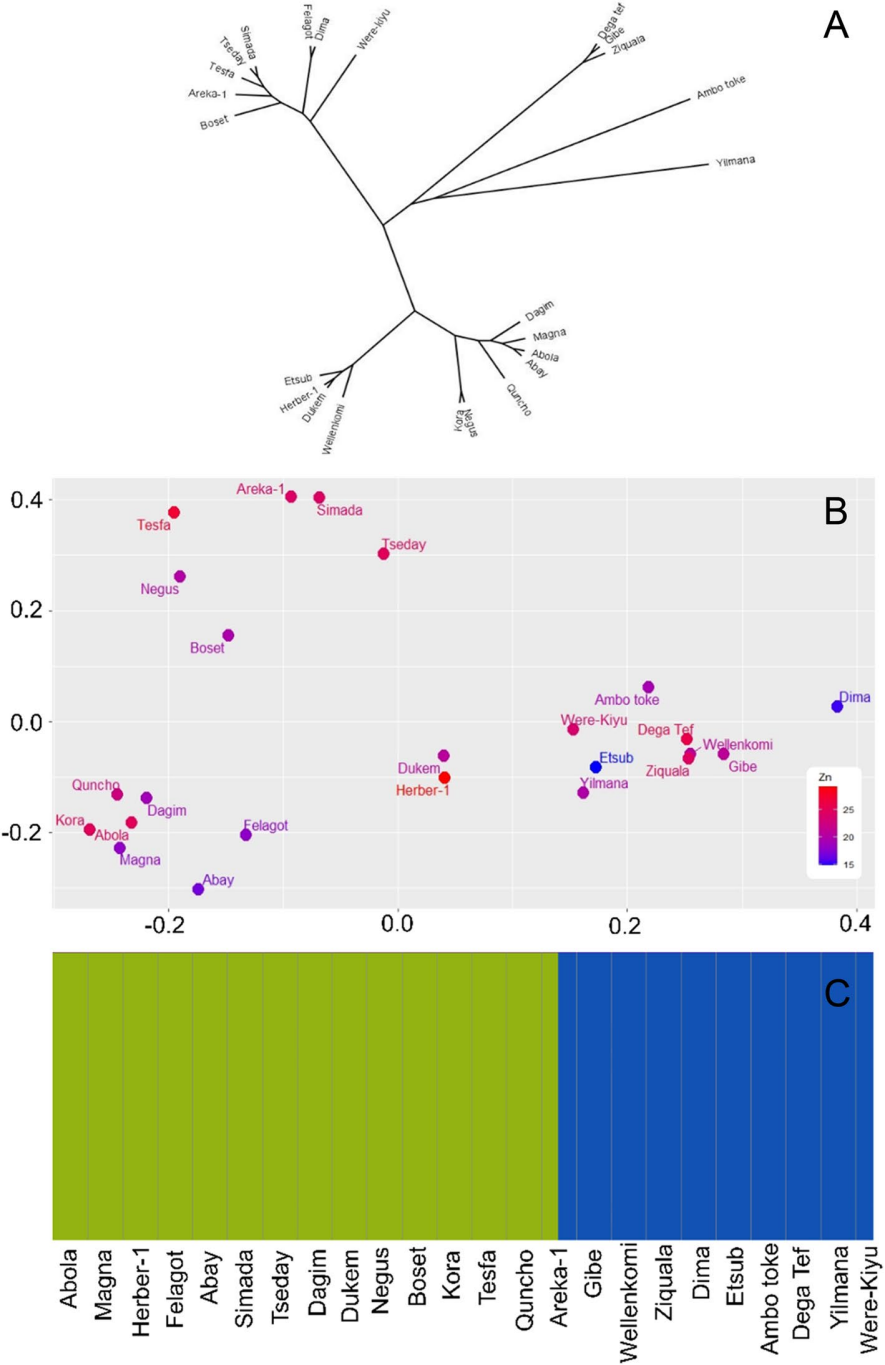
Population characteristics of the 24 sequenced tef varieties constructed using genome wide SNPs. **A** Phylogenetic tree, **B** PCA plot overlayed with grain Zn content, and **C** Population structure predicted using FastStructure

To understand how the 24 tef varieties relate to each other a phylogenetic tree analysis (Fig. 5A), a principal component analysis (PCA; Fig. 5B), and a structure analysis (Fig. 5C) were carried out using the genome wide SNP data. The phylogenetic tree and PCA separated the 24 tef varieties into 3 groups, however the structure analysis returned two groupings. In general, the tef varieties fell into similar groupings when comparing the phylogenetic tree and PCA, the exception being the varieties Negus and Kora, which fell on the same branch of the tree but in distinct PCA groups.

### Genetic variation underlying differences in Zn levels

Zn concentrations were overlaid on the PCA to see if a relationship between flour Zn levels and the tef variety groupings could be identified (Fig. 5B). As no specific association between genetic grouping of tef varieties and Zn concentration were apparent, we chose to look at the genetic variation in specific gene families involved in Zn transport. Two gene families were selected. The ZIP (Zinc Iron Permease) family are involved in uptake of Zn from the soil and the HMA (Heavy metal associated) family of transporters are involved in movement of Zn from roots to seeds.

### Zinc Iron permease transporter family in tef

To identify putative ZIP family members the gene models of the *Eragrostis tef* var. Dabbi sequence in Ensembl plants was searched using the PFAM domain PF02535 and Interpro ID IPR003689. This revealed 32 predicted genes in tef which contained these protein domains. As tef is a tetraploid species these 32 potential ZIP family members is comparable to the 15 in *Arabidopsis* and 17 in rice [32, 54]. However, some of the predicted genes could be pseudogenes. Only six of the 32 coding regions identified have a good ATG start codon, and two of these six putative ZIP transporters lacked a stop codon. However, as this incomplete sequence data could be due to gaps in the reference var. Dabbi tef sequence we used all 32 putative ZIP sequences in subsequent analyses.

**Fig. 6:**
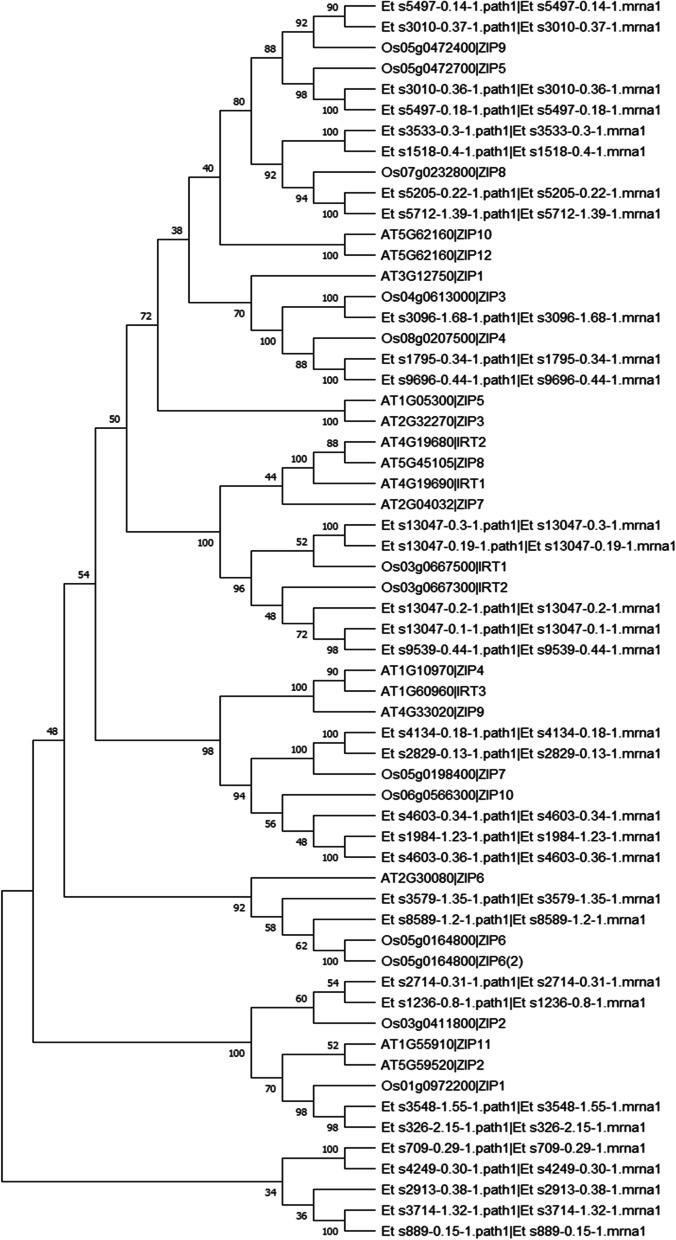
Phylogenetic tree of ZIP transporters. Shown is a maximum likelihood tree of Arabidopsis (At), rice (Os) and tef (Et) ZIP amino acid sequences. Values at each node are the calculated boot strap values from 50 iterations of the tree

Phylogenetic analysis of the translated, amino acid sequences of the 32 tef ZIP transporters was performed with members of the ZIP families from rice and *Arabidopsis* Fig. 6). Tef ZIP proteins showed closer linkages with rice ZIP proteins compared to *Arabidopsis*, both tef and rice being monocots. In most cases there were two tef genes for every rice ZIP gene, which is expected as tef is a tetraploid species, but not all 32 tef ZIP genes showed clear associations with genes found in rice or *Arabidopsis*, suggesting some divergence. Rice ZIP genes associated with more than two tef genes included OsZIP8, which was associated with four tef genes. Only one of the 32 tef ZIP genes localized with OsZIP3, while three tef genes colocalized with OsIRT2, as well as OsZIP10.

### Heavy metal associated transporter family in tef

**Fig. 7:**
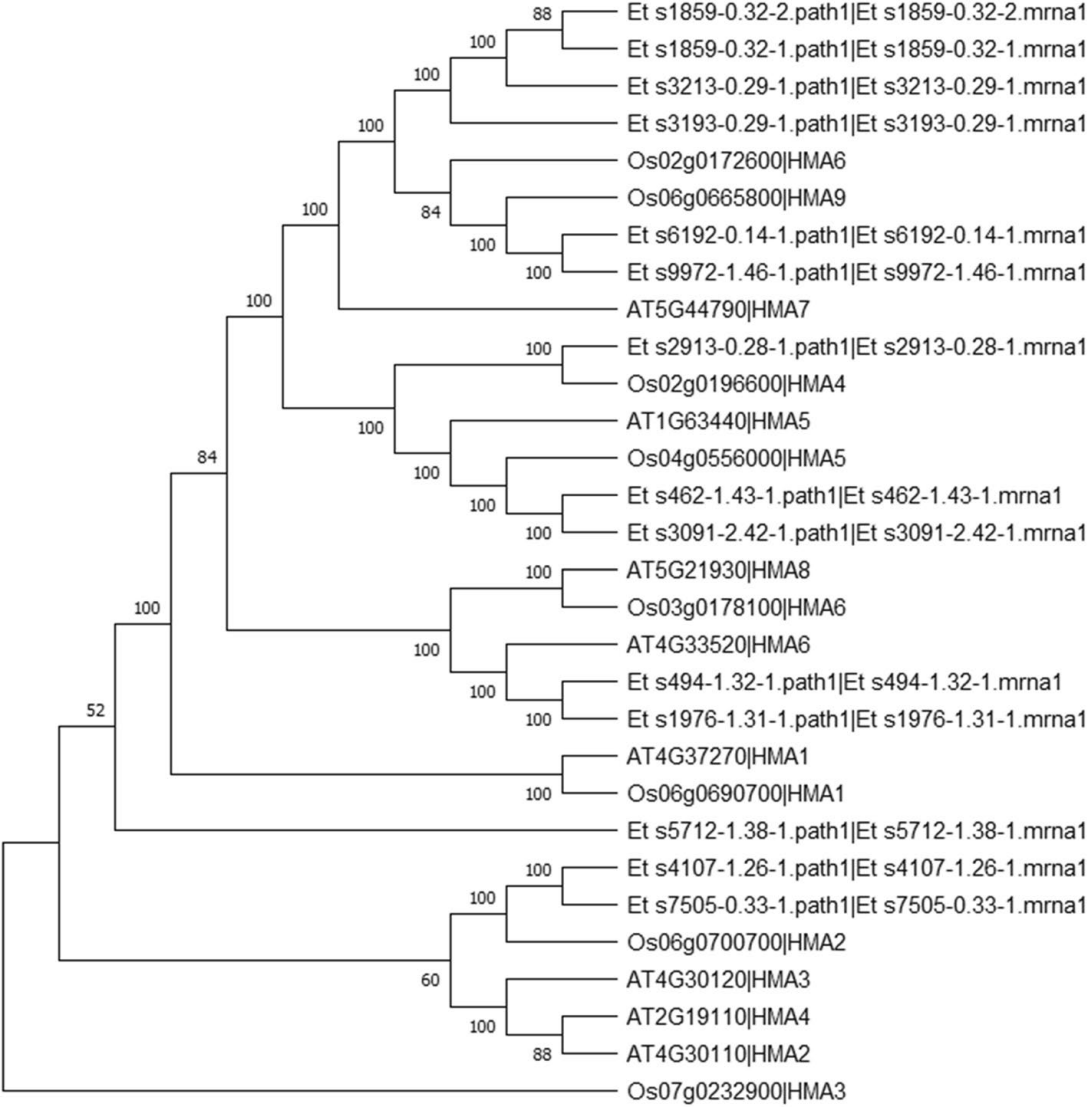
Phylogenetic tree of HMA transporters. Shown is a maximum likelihood tree of Arabidopsis (At), rice (Os) and tef (Et) HMA amino acid sequences. Values at each node are the calculated boot strap values from 50 iterations of the tree

To identify putative HMA family members the gene models of the *Eragrostis tef* var. Dabbi sequence in Ensembl plants was searched using the Interpro domain IPR027256. Fourteen HMA proteins were identified in tef compared to eight in *Arabidopsis* and nine in rice. Phylogenetic analysis of the translated, amino acid sequences of the 14 tef HMA transporters was performed with members of the HMA families from rice and *Arabidopsis* (Fig. 7). As 14 tef HMA genes was less than expected for this tetraploid species, based on conservation of each of the eight core genes found in both rice and *Arabidopsis,* the tef Dabbi reference sequence may therefore be incomplete with regards to this family of Zn transporters. In addition, five of the 14 predicted HMA genes in tef did not contain an ATG start codon, suggesting that some sequence maybe missing, or that the gene models are incorrect. Only four rice HMA proteins, OsHMA2, OsHMA5, OsHMA6 and OsHMA9, had two clear homologues within the predicted tef HMA genes. However, no clear homologue for the rice OsHMA3 gene, a major gene involved in Cd tolerance and sequestration [55], was detected.

### Identification of variants in ZIP and HMA proteins

Numerous SNPs were found in both the ZIP and HMA tef families of transporters (Additional files 6, Additional file 7). In the 32 *EtZIP* genes a total of 355 variants were identified in the coding regions relative to the reference var. Dabbi, this included frame shift mutations in twelve of the 32 genes (3.4% of the total mutations) (Additional file 6). Most of the variants were located in an intron (41.4%), followed by synonymous variants in the coding region (22.5%), and then by non-synonymous mutations that result in a change in the amino acid sequence (14.6%). Many of the variants were conserved between the varieties, including a frameshift in both homologues of OsZIP9, with most of the varieties containing the mutated/truncated form of each gene.

In *EtHMA* transporters 298 variants were identified relative to var. Dabbi (Additional file 7). Most of the variants were again found in introns or represented synonymous mutations in the coding sequence (48 and 16.4%, respectively). Only two of the 14 tef HMA genes had frame shift variants. This included the predicted gene loci Et_s3091–2.42-1.mrna1 and Et_s3193–0.29-1.mrna1. The third most common variants were non-synonymous mutations in the protein coding region (12.4%).

### Genetic variation underlying differences in phenolic compounds

**Fig. 8:**
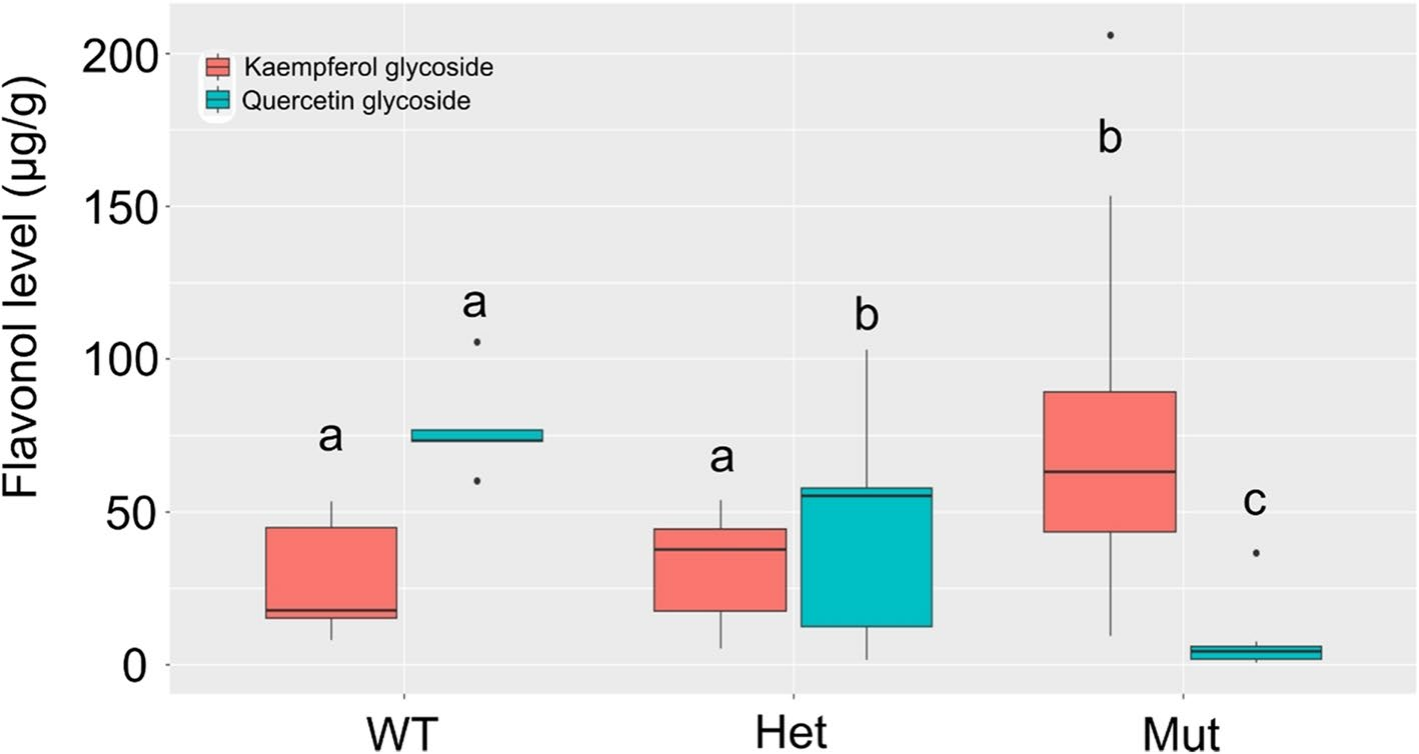
Haplotype analysis of a F3’H gene in tef. Kaempferol glycoside and quercetin glycoside levels in tef flours given the genotype of the T → G mutation in the second intron of Et_s3159-0.29-1.mrna1. A different letter represents a significant difference (*p* val < 0.05) between genotypes for either kaempferol glycoside or quercetin glycoside

Flavonoid 3′-hydroxylase (F3’H) is a key enzyme in the conversion of dihydrokaempferol to dihydroquercetin [53, 56]. To determine whether F3’H in tef was responsible for the differences seen between tef varieties in kaempferol glycoside and quercetin glycoside levels the amino acid sequence of the rice F3’H gene (Os10g0320100) was used to identify possible orthologs in tef. Using an e-value cut off of 1e-10 five putative orthologs of OsF3’H were identified in the tef reference sequence of var. Dabbi: Et_s9738-1.8-1.mrna1, Et_s9399-0.5-1.mrna1, Et_s3159-0.29-1.mrna1, Et_s6352-0.10-1.mrna1 and Et_s15942-0.0-1.mrna1 (Suppl. Fig. 2). Alignment of the amino acid sequences of these five tef genes to their rice putative orthologs showed 58.74 to 78.57% identity. The two tef genes Et_s3159-0.29-1.mrna1 and Et_s15942-0.0-1.mrna1 clustered most closely with Os10g0320100 and were subject to further analysis. SNPs were identified between Et_s3159-0.29-1.mrna1 and Et_s15942-0.0-1.mrna1. Correlations between these SNPs and the levels of kaempferol glycoside and quercetin glycoside in the 24 tef varieties revealed a SNP in Et_s3159–0.29-1.mrna1 that was strongly correlated with kaempferol glycoside and quercetin glycoside levels (*p* val < 0.001). This SNP resulted in a T → G substitution in the second intron of Et_s3159–0.29-1.mrna1, and therefore does not directly change the coding sequence of the gene (Suppl. Fig. 2). Tef varieties containing the wild-type (WT) “T” allele in the homozygous state had lower levels of kaempferol glycoside than tef varieties containing the mutant “G” SNP, and visa verse, while varieties with a heterozygous SNP had intermediary levels of the two glycosides (Fig. 8). No further associations were found between the other phenolic compounds measured and SNPs in any of the F3’H-type genes in tef.

## Discussion

Tef is a staple crop for many Ethiopians, however, due to naturally low Zn levels in soils on which it is grown the grain does not meet the Zn needs of those who consume it [57]. Zn is essential for a healthy immune system and stimulates the activity of many different enzymes. The low level of Zn in tef has therefore led to many Ethiopian children suffering from Zn deficiency [58]. Fe is also an essential element, required for normal blood cell function [59], but has a low bioavailability, the small intestine not readily absorbing large amounts of Fe. However, a number of compounds have been shown to influence Fe absorption in the human gut, including phytate, kaempferol and quercetin [60–62].

To understand the breeding potential of tef for Zn and Fe content, as well as several other nutritional factors, grain from 24 tef varieties were assayed for a range of nutritional traits. Analysis of these 24 varieties showed significant variation in all the micronutrients tested, with a twofold difference in the amount of Zn being observed. However, even var. Heber-1, with 29.2 mg/kg of Zn, had levels below the recommended 40 mg/kg of Zn in flour [8]. This may be rectified by Zn fertilization of the soil, but future work would be required to understand how each variety responds to Zn soil supplements, given the complexity of Zn uptake and transportation to the grain.

No correlation was found between Zn and Fe levels in the grains, and is most likely due to different regulatory mechanisms and transporters involved in the movement of each ion through the plant [18, 30]. The inability to identify a homologue of Os*HMA3*, a gene associated with Cd tolerance and sequestration in rice, may also suggest an underlying cause for some of the high levels of Cd seen in the 24 tef flours. However, it should also be noted that other putative tef genes, including a homolog of *OsZIP1* (Et_s3548–1.55-1.mrna1) which is involved in efflux of excess heavy metals, was one of the twelve tef genes with a frame shift variant present in all the tef varieties. This would suggest that multiple genes which sequester Cd in the roots, and keep it away from the grain, may not exist in tef or are non-functional in the modern tef varieties assayed in this study [63].

Sequencing of the 24 tef varieties enabled the identification of several variants which could be used to develop markers for future breeding. Overall, there was a large amount of genetic variation between the varieties compared to the reference var. Dabbi. Although most of these variants were in non-coding regions or synonymous mutations, as seen in the analysis of the ZIP and HMA transporter families. The variation observed is not unexpected as similar levels of variation have been observed in a core set of rice varieties which were recently sequenced [64].

In rice, double mutants of *OsZIP5* and *OsZIP9* have shown severe Zn deficiency symptoms, suggesting these two genes are the major route for Zn into the rice plant [23, 24]. However, mutants of either gene in isolation does not show major effects on Zn uptake. Mutations in two homologues of *OsZIP9,* including frame shifts, as well as a frame shift variant in a homologue of *OsZIP5* in some varieties, would suggest that the route of Zn into tef, while compromised by these mutations, is not wholly dependent on the homologues of *OsZIP5* and *OsZIP9*. Other transporters maybe the major route for Zn into tef plants. These other routes might also contribute to the high levels of Cd seen in many of the tef varieties. It has been shown that genes involved in Mn and Fe uptake can also transport Cd in vivo whereas high affinity Zn transporters do not appear to have this capability [17, 25, 29, 65, 66].

Comparison of other nutritional traits, including nitrogen (as a proxy for protein), phytate and a number of phenolic compounds, also showed significant variation between the 24 tef varieties. The variation in the phenolic profiles is of importance as some phenolics have been found to alter Fe bioavailability and may therefore present a more effective strategy than Fe fortification for enhancing nutritional outcomes. It is often reported that phytate inhibits Fe bioavailability, so the negative correlation between Fe and phytate levels in tef could prove beneficial. While future research is required to determine whether elevated levels of phenolics that stimulate and inhibit Fe uptake in the human gut can help alleviate the anaemia seen in Ethiopian children [67], the significance of the SNP in the tef F3’H gene Et_s3159-0.29-1.mrna1, which explains a large proportion of the variation in kaempferol glycoside and quercetin glycoside levels, suggest this might be a breeding target to improve Fe bioavailability in tef, as kaempferol glycoside is known to promote Fe absorption while quercetin glycoside inhibits Fe absorption in cell assays [60]. In addition, phenolic compounds have also been found to influence other agronomic traits, including tolerance to both biotic and abiotic stress [16, 60, 61, 68].

With the considerable variation seen for the nutritional traits assessed in this study breeders are in a good position to breed for enhanced nutritional value in tef. The genomic sequence information collected can be used to identify and develop markers linked to target genes and traits. We have yet to test the heritability of these traits and their stability over growing locations and seasons, but marker-assisted selection, particularly within target genes, can now provide a feasible approach to breed for these nutritional traits in tef.

## Conclusions

For many subsistence Ethiopian farming families tef is a major crop and source of calories. Yet, as shown in this study, levels of Zn are usually below recommended levels, while levels of Cd exceed EU limits. However, considerable phenotypic and genetic variation for a range of nutritional traits and the genes regulating their levels in planta, exists. This provides considerable potential to determine the relationship between these nutrition phenotypes and identify allelic variants that would allow breeding of new tef varieties with optimal nutritional potential.

## Supplementary Information

**Additional file 1.** Twenty-four teff varieites use in study.

**Additional file 2.** Average elemental concentrations of three replicates of teff flour acid digested and measured by ICP-MS, phytate, and N levels measured by Dumas.

**Additional file 3.** Average amounts of 12 phenolic compounds found in 24 teff varieties (μg/g flour) and 7 phenolics not found in significant quantities.

**Additional file 4.** Correlations between phenolic compunds, phytate and Fe.

**Additional file 5.** SNP and INDEL variants relative to var. Dabbi for each of the 24 teff varieties sequenced.

**Additional file 6.** Variants identified in the coding regions of teff ZIP type transporters.

**Additional file 7.** Variants identified in the coding regions of teff HMA type transporters.

**Additional file 8.** Genotype of SNP in the second intron of Et_s3159-0.29-1.mrna1 in the varieties tested. Where WT is a T and mut is a G.

**Additional file 9: Fig. 1**. Significant correlations between the elemental concentrations in 24 teff flours. Size of the circle represents the significance level, with the larger the circle the lower the *p* value. All circles represent a *p* value less than 0.05. Scale on the left is the range of correlations (r). **Fig. 2**. Phylogenetic tree showing the relationship between the five teff homologues of the rice F3’H gene Os10t0320100–01.

## Data Availability

Sequencing Data is available at the ENA under ArrayExpress accession E-MTAB-8827.

## Abbreviations

N: Nitrogen
Zn: Zinc
P: Phosphate
Fe: Iron
Cd: Cadmium
Pb: Lead
Ca: Calcium
S: Sulphur
K: Potassium
Mg: Magnesium
Mn: Manganese
Mo: Molybdenum
Se: Selenium
Ti: Titanium
Al: Aluminium
Si: Silicon
HPLC: High-performance liquid chromatography
PDA: Photo Diode Array
HCl: Hydrochloric acid
Vcf: Variant Call Format
EU: European Union
MTP: Metal Tolerance Protein
ZIP: Zinc Iron Permease
HMA: Heavy Metal Associated
NRAMP: Natural Resistance-Associated Macrophage Proteins
SNP: Single Nucleotide Polymorphism
INDEL: Insertion or Deletion
ICP-MS: Inductively Coupled Plasma Mass Spectrometry
PCA: Principal Component Analysis
ENA: European Nucleotide Archive
