# Supplementary material for: Nutritional and genetic variation in a core set of Ethiopian Tef (Eragrostis tef) varieties
Source: BMC Plant Biol. 2022 Apr 28;22:220. doi: 10.1186/s12870-022-03595-9 (PMC9047342; doi:10.1186/s12870-022-03595-9)
Supplement: Supplementary file 9 — Additional file 9: Fig. 1. Significant correlations between the elemental concentrations in 24 teff flours. Size of the circle represents the significance level, with the larger the circle the lower the p value. All circles represent a p value less than 0.05. Scale on the left is the range of correlations (r). Fig. 2. Phylogenetic tree showing the relationship between the five teff homologues of the rice F3’H gene Os10t0320100–01. [file 12870_2022_3595_MOESM9_ESM.docx]

Suppl. Figures


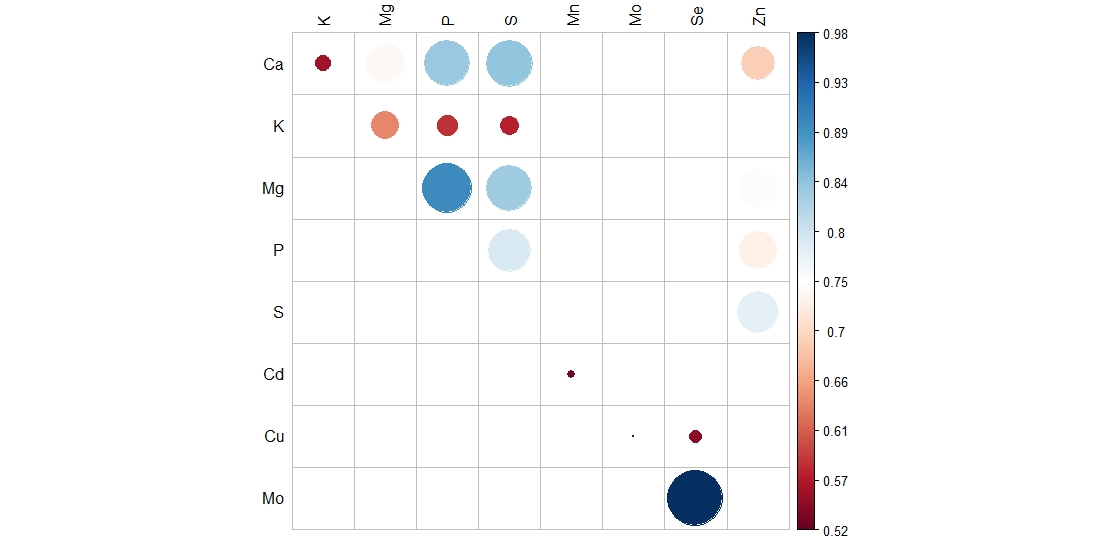


Suppl. Figure 1: Significant correlations between the elemental concentrations in 24 teff flours. Size of the circle represents the significance level, with the larger the circle the lower the p value. All circles represent a p value less than 0.05. Scale on the left is the range of correlations (r).


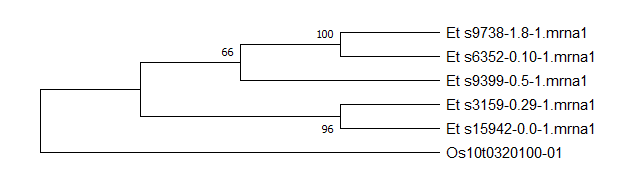


Suppl. Figure 2: Phylogenetic tree showing the relationship between the five teff homologues of the rice F3’H gene Os10t0320100-01
